# The impact of ethnicity and intra-pancreatic fat on the postprandial metabolome response to whey protein in overweight Asian Chinese and European Caucasian women with prediabetes

**DOI:** 10.3389/fcdhc.2022.980856

**Published:** 2022-10-14

**Authors:** Aidan Joblin-Mills, Zhanxuan Wu, Karl Fraser, Beatrix Jones, Wilson Yip, Jia Jiet Lim, Louise Lu, Ivana Sequeira, Sally Poppitt

**Affiliations:** ^1^ Food Chemistry and Structure Team, Agresearch, Palmerston North, New Zealand; ^2^ High-Value Nutrition, National Science Challenge, Auckland, New Zealand; ^3^ School of Food and Nutrition, Massey University, Palmerston North, New Zealand; ^4^ Department of Statistics, University of Auckland, Auckland, New Zealand; ^5^ Human Nutrition Unit, School of Biological Sciences, University of Auckland, Auckland, New Zealand

**Keywords:** prediabetes, whey protein isolate, ethnicity, intra-pancreatic fat deposition, metabolomics, machine learning, pathway enrichment, network topology

## Abstract

The “Thin on the Outside Fat on the Inside” TOFI_Asia study found Asian Chinese to be more susceptible to Type 2 Diabetes (T2D) compared to European Caucasians matched for gender and body mass index (BMI). This was influenced by degree of visceral adipose deposition and ectopic fat accumulation in key organs, including liver and pancreas, leading to altered fasting plasma glucose, insulin resistance, and differences in plasma lipid and metabolite profiles. It remains unclear how intra-pancreatic fat deposition (IPFD) impacts TOFI phenotype-related T2D risk factors associated with Asian Chinese. Cow’s milk whey protein isolate (WPI) is an insulin secretagogue which can suppress hyperglycemia in prediabetes. In this dietary intervention, we used untargeted metabolomics to characterize the postprandial WPI response in 24 overweight women with prediabetes. Participants were classified by ethnicity (Asian Chinese, n=12; European Caucasian, n=12) and IPFD (low IPFD < 4.66%, n=10; high IPFD ≥ 4.66%, n=10). Using a cross-over design participants were randomized to consume three WPI beverages on separate occasions; 0 g (water control), 12.5 g (low protein, LP) and 50 g (high protein, HP), consumed when fasted. An exclusion pipeline for isolating metabolites with temporal (T_0-240mins_) WPI responses was implemented, and a support vector machine-recursive feature elimination (SVM-RFE) algorithm was used to model relevant metabolites by ethnicity and IPFD classes. Metabolic network analysis identified glycine as a central hub in both ethnicity and IPFD WPI response networks. A depletion of glycine relative to WPI concentration was detected in Chinese and high IPFD participants independent of BMI. Urea cycle metabolites were highly represented among the ethnicity WPI metabolome model, implicating a dysregulation in ammonia and nitrogen metabolism among Chinese participants. Uric acid and purine synthesis pathways were enriched within the high IPFD cohort’s WPI metabolome response, implicating adipogenesis and insulin resistance pathways. In conclusion, the discrimination of ethnicity from WPI metabolome profiles was a stronger prediction model than IPFD in overweight women with prediabetes. Each models’ discriminatory metabolites enriched different metabolic pathways that help to further characterize prediabetes in Asian Chinese women and women with increased IPFD, independently.

## Introduction

The prevalence of type 2 diabetes (T2D) in China has increased drastically in recent decades, reaching epidemic proportions (1). As mainland China represents the highest number of T2D and prediabetic cases worldwide, most concerning to the population is the increasing prevalence in young and lean adults, a worse profile when compared to, for example, more resilient European Caucasians (2, 3). The susceptibility of Asian Chinese to T2D can be attributed to both genetic and lifestyle risk factors, with decreased exercise and westernized diets important (4, 5). Likely to play a role in exacerbating T2D onset and metabolic syndrome (6, 7) is the preferential accumulation of both visceral adipose tissue (VAT) and ectopic organ fat, far more so than permissive subcutaneous adipose tissue (SAT).

A high VAT+organ fat to SAT ratio in outwardly lean individuals has been termed the “Thin on the Outside, Fat on the Inside” (TOFI) phenotype, and may help to explain the high T2D risk among Asian countries relative to other parts of the world (8, 9). A high VAT/SAT ratio among Asian cohorts has previously been associated with hyperglycemia, hyperinsulinemia and/or insulin resistance, high blood pressure, and increased levels of plasma uric acid and triglycerides (TGs), regardless of body mass index (BMI) and/or a diabetic diagnosis (10–12). The accumulation of SAT in overweight individuals has been proposed to provide a beneficial sink for free fatty acids and TGs, reducing the exposure of organs to lipotoxic stress (13). Several authors have proposed that when the lipid storage capacity of SAT becomes oversaturated, individuals may be predisposed to excess VAT and increased accumulation of ectopic fat in skeletal muscle, epicardial tissue, liver and pancreas (14–16). Why some individuals are more susceptible to ectopic fat accumulation has not yet been established.

Magnetic resonance imaging and spectroscopy (MRI and MRS) shows differences in SAT, VAT, and ectopic fat depots to be poorly identified using standard anthropometry techniques and total body fat assessments (9, 17). A previous study from our laboratory conducted by Wu et al. demonstrated that SAT, VAT, pancreas and liver fat could be characterized through untargeted lipidomics and metabolomics methods (18). Using MRI and MRS to characterize body fat depots in healthy and pre-diabetic Caucasian and Chinese women in the TOFI_Asia study, we subsequently identified a set of metabolomic markers that could successfully predict the fat levels of high VAT/SAT ratios, intra-pancreatic and intra-liver fat depots, with greater predication accuracy (cum R^2^) than typical clinical markers, cardiovascular disease (CVD) risk factors, and anthropometric measurements. Using partial least squares discriminative analysis (PLS-DA) we also identified a clear and robust separation in lipid and polar metabolite profiles that characterized the two ethnic cohorts (19).

Whilst intra-pancreatic fat deposition (IPFD) has been linked to suppressed insulin secretion in participants with impaired glucose tolerance (IGT) and impaired fasting glycaemia (IFG) (20), a recent review has reported a series of findings showing low level IPFD to be common even in metabolically healthy individuals (21). The authors propose that a clearer distinction between fatty pancreas disease (FPD) and the non-disease related IPFD is required. A large-scale MRI study conducted by Wong et al. assessed FPD in a cohort of 685 Hong Kong residents (≥18 years of age), using the 95th percentile of IPFD in individuals without metabolic syndrome or alcohol abuse as a cutoff, and proposed 10.4% as the IPFD cut point for FPD. However, there is no international standard for MR-assessed IPFD established at the time of writing. Their cutoff point identified 16.1% prevalence of FPD in the Asian Chinese cohort (22). With such a high tendency for Asian Chinese to accumulate VAT/SAT and develop FPD, the role of IPFD in pathogenesis of T2D remains unclear. IPFD may be quite widespread throughout the world’s population (21). It raises the question of whether IPFD is part of the TOFI phenotype, and whether it plays a significant role in T2D progression from prediabetes, or whether other factors associated with the Asian Chinese ethnicity are more pertinent.

Several dietary intervention studies investigating prevention of T2D, weight loss and postprandial satiety have shown promising results for dairy products, in particular the whey protein fraction (23–25). Rich in essential amino acids (EAAs), non-essential amino acids (NEAAs) and essential branch-chain amino acids (BCAAs), whey protein isolate (WPI) can decrease postprandial hyperglycemia and promote insulin secretion in both healthy and diabetic individuals (26, 27). We questioned whether differences in postprandial response to WPI would be detectable using a broader untargeted metabolomics platform, to compare prediabetic (raised fasting plasma glucose, FPG, 5.6–6.9 mmol/L) European Caucasian and Asian Chinese women with varying degrees of IPFD.

Due to the natural variability that exists among cohorts, identifying phenotypic biomarkers from large-scale omics datasets can be a difficult task. One method metabolomic researchers have begun using is support vector machine-recursive feature elimination (SVM-RFE) algorithm (28). SVM-RFE machine learning is an optimal method for identifying phenotypic features from small cohort studies, due to the implementation of a SVM kernel trick; which projects variables from a 2-dimensional space to a 3-dimensional space, providing more options for optimizing decision boundary parameters for the classification of a phenotype (29). Use of SVM with RFE not only allows for optimal discrimination of different classes in a model, but also identifies the important variables contributing towards the classification model, complementing traditional multivariate analysis (30). This has proven to be an effective method for classifying different cancers from large scale genomic datasets (31, 32).

With the development and curation of open-source databases such as the Human Metabolome database (HMDB) and Kyoto Encyclopedia of Genes and Genomes (KEGG) (33, 34), the detection and annotation of hundreds of metabolites *via* hydrophilic interaction chromatography tandem mass spectrometry (HILIC–MS) facilitates the use of more holistic approaches to data processing, such as network topology and bio-ontology enrichment analysis’ (35–37). These bioinformatic tools provide researchers with a method for discerning biological relevancy from data complexity. Thus, we aimed to model the WPI metabolome response firstly for European Caucasian and Asian Chinese participants, and secondly for participants with lower and higher IPFD than the median value for our current cohort (4.66% IPFD), to determine their impact on metabolic markers and metabolic pathways associated with prediabetes. Notably the IPFD cut point was comparable with that of Singh and colleagues in their 2017 systematic review and meta-analysis (38).

## Materials and methods

### Study design

The presented work is a continuation of previously reported TOFI_Asia studies (7, 18). The recruitment procedures, study design, WPI composition, participant characteristics, appetite biomarkers and gluco-corticoid hormone measurements have been summarized previously by Lim et al. (39). In brief, this was an acute, randomized, three treatment cross-over study which investigated the postprandial WPI response of 12 Asian Chinese females and 12 European Caucasian females, aged 20–69 years and BMI 19.6–36.8 kg/m^2^. At the time of screening, all participants had prediabetes based on ADA criteria, with raised FPG of 5.6–6.9 mmol/L (40). Magnetic resonance imaging (MRI) was used to quantify pancreatic fat in 20 participants, as detailed by Wu et al. (18). low and high IPFD were defined as < and ≥ the cohort median of 4.66%, respectively. Each participant attended the Human Nutrition Unit (HNU), University of Auckland, New Zealand for three study visits over a three-week duration, with a minimum seven-day wash-out period. At each visit, a fasted baseline (T = 0 min) plasma sample was collected. Following consumption of the 280 mL test drinks comprising 0 g (water control, WC), 12.5 g (low protein, LP) and 50 g (high protein, HP) WPI, postprandial plasma samples were collected *via* a venous cannula at T = 30, 60, 120 and 240 min. No other foods or beverages were consumed during the study morning and participants followed a sedentary protocol.

### Metabolomics procedures

#### Sample preparation

Blood samples were stored at -80°C and batch analysed at the end of the study. For each sample, 100 μL plasma was mixed with 800 μL pre-chilled (-20°C) CHCl_3_:MeOH (50:50, v/v), agitated for 30 s and placed in a -20°C freezer for 60 min to allow protein precipitation. 400 μL milliQ water was subsequently added to each sample, agitated for 30 s and centrifuged at 11,000 rpm at 4°C for 10 min. From each biphasic separation, 200 μL of the upper aqueous layer was transferred to 2 mL micro-centrifuges and dried under a nitrogen stream before being stored at -80°C. To account for batch-to-batch variations, pooled quality control (QC) samples were prepared by pooling aliquots from each sample into a clean glass vial and stored at -80°C (41). Pooled samples were combined from each batch and dispensed into separate 200 µl aliquots for drying under a nitrogen stream and -80°C storage. Dried polar extracts were reconstituted in 200 μL acetonitrile:H_2_O (50:50, v/v) before injection (18).

#### Liquid chromatography tandem mass spectrometry conditions

Polar metabolites were analysed with an Accela 1250 quaternary UHPLC pump coupled to an Exactive Orbitrap mass spectrometry (Thermo Fisher Scientific, USA). Chromatographic separation was carried out at 25°C on a SeQuant^®^ ZIC^®^-pHILIC 5 µm 2.1 mm × 100 mm column (Merck, Darmstadt, Germany) using the following solvent system: A = 10 mM ammonium formate in milliQ water, B = 0.1% formic acid in acetonitrile at a gradient program flow rate of 250 µL/min: 3–3% A (0.0–1.0 min), 3–30% A (1.0–12.0 min), 30–90% A (12.0– 14.5 min). 90% A was maintained for 3.5 min followed by re-equilibration with 3% A for 7 min. An injection volume of 2 µL was used. The electrospray probe was operated at room temperature (25°C) to avoid degradation of thermally labile compounds. External mass calibration of the Orbitrap prior to sample analysis was performed by the flow injection of the calibration mix solution according to manufacturer’s instruction. High resolution (25,000) data were acquired by full scan (m/z 55 to 1100) with a source voltage of 4000 V for both ESI + and ESI - ion modes. A capillary temperature of 325°C was set, and sheath, auxiliary, and sweep gas flow rates of 40, 10, and five arbitrary units were applied, respectively (42).

#### Peak processing

Raw datafiles were converted to mzXML format using ProteoWizard tool MSconvert (v 3.0.1818). Open mzXML data files were pre-processed for features by untargeted peak filtering, peak alignment, and peak filling parameters with the XCMS package (v3.0.2) in the R programming environment (v3.2.2) (43, 44). Features not detected in 100% of the QC samples were excluded from the analysis and resulting extracted ion chromatograms were manually examined to filter poorly integrated peaks generated by the diffreport function. Signal drift and batch effects were corrected for by LOESS algorithm in the online W4M Galaxy environment, and feature filtering with a < 30% coefficient of variation limit among QC samples was applied (45, 46).

### Feature exclusion pipeline

Before pre-processing, a K-nearest neighbours’ algorithm was used to impute values for two missing samples. A Shapiro-Wilk normality test was performed over the dataset and features with p-value ≤ 0.05 were log transformed to reduce skewed distributions. The resulting data set was mean centred, and summary scaled to normalise and standardise value ranges (47). To determine likely features impacted by WPI intake, linear mixed-effect (LME) modelling with 10,000 permutations was performed with Meal, Time, Meal*Time, Age and BMI as fixed effects, and participants ID’s as random effects (48). LME modelling was performed using the nlme package in the R environment. Benjamini-Hochberg false discovery adjustments were applied to p-values and resulting features with q-values ≥ 0.05 were filtered out.

To further filter out LME false positives, incremental area-under-the-curve (iAUC) calculations were performed against remaining features using the trapezoidal method in Graphpad Prism (v9.0.0); values were obtained for each meal per participant using the mean of each feature at T=0 as a baseline and ignoring peaks less than 75% of the height from minimum to maximum Y, and peaks defined by fewer than three adjacent time points. Feature net area values were subsequently used to measure fold-change (FC) between each WPI concentration and water control in MetaboAnalyst (v5.0.0) (49, 50). Features with a significant log^2^ FC for the comparison of 12.5 g/0 g WPI alone were removed and remaining features with a significant log^2^ FC for both WPI meal comparisons and features significant for the 50 g/0 g WPI comparison alone, were shortlisted for modelling.

### Machine learning and multivariate modelling

#### Support vector machine-recursive feature elimination and cross-validation

Support vector machine–recursive feature elimination (SVM-RFE) procedures were implemented using the Github repository code provided by John Colby (51), and the e1071 package in R environment. Linear kernel SVM was used to rank all AUC-log^2^FC features by weight across a 10-fold cross-validation (CV) set for the classification of ethnicity (Caucasian and Chinese, n=24) and pancreatic fat (IPFD_Low and IPFD_High, n=20) as postprandial WPI metabolome models (31). Feature ranks were averaged across all training set folds and the lowest weights were removed by “multiple RFE”, wherein reducing the feature total by half before introducing traditional “one-by-one” SVM-RFE (32). Final ranking scores for top features were presented as averages across all training folds per model. To obtain generalized error estimates for testing folds, a radial basis function (RBF) kernel SVM was first applied to training folds with optimal tuning of the SVM hyperparameters (Cost and Gamma combinations) by internal 10-fold CV. Optimal parameters were used to train the SVM of each training fold before predicting corresponding test folds to calculate generalized error estimates (52). All testing fold generalization error estimates were averaged, and the process repeated with varying numbers of top features as input at each iteration to determine optimal number of features for a given classification model. For each model matrix, a comparative confusion (dummy) matrix was created by reassigning each feature-columns *y* values to a random class ID using the Kutools package in Excel (53). Ethnicity and IPFD dummy matrices were subject to all SVM-RFE procedures to calculate average feature ranks and generalized error estimates for comparison to respective query models. Top 20 features were annotated as metabolites and presented with SVM-RFE average ranking value and their respective Meal*Time LME interaction significance.

#### Multivariate analysis

All multivariate analysis was performed in SIMCA software version 16 (Sartorius, Umeå Sweden). Principle component analysis (PCA) and partial least squares-discriminant analysis (PLS-DA) was applied to evaluate each classification model’s performance through mSVM-RFE ranked features across all plasma samples (i.e Time and Meal). PLS-DA models were subject to 100-fold permutations to evaluate separation performances and visualized (30).

### Metabolite annotations

A subset of positive and negative mode features were annotated using accurate mass and retention time matching to an in-house library of authentic standards. These were used as a quality control measure for annotating features with the metID package (GitHub) in R (54). Features were subsequently annotated using metID’s hilic 0.0.2 database with accurate mass and a retention time window shift of 420 s as calculated from initial in-house library matching. Features that lacked an annotation had their molecular and co-eluting ions manually inspected for pseudo MS2 fragmentation patterns created from in-source fragmentation. Pseudo fragments and chemometric features (e.g., isotopes, multiple adducts) representing the same metabolites were annotated accordingly. Remaining features were annotated using the online Human Metabolome Database (HMDB) using an m/z ppm error of 15 for positive and negative ion modes (33).

### Metabolic network analysis and pathway enrichment

Machine learning and multivariate processing of metabolomics features determines the weight of each metabolite through vector values alone, which can be advantageous when characterizing metabolites with unknown annotations, but becomes restricted in application without consideration of a given metabolite’s biological ontology (55, 56). To determine the relevance of Ethnicity and IPFD model metabolites to established networks and metabolic pathways, the KEGG IDs of top-ranking metabolites for each query model were subject to network construction and topological analysis using the MetScape plugin (v3.1.3) within Cytoscape (v3.1.3) (57), and pathway enrichment with Metabolite Set Enrichment Analysis (MSEA) in MetaboAnalyst (58). Each query model was visualized as a network through Metscapes pathway-based network build function, and topological parameters were extracted using the network analyzer tool (59). To identify the most important metabolites of the network, a relative betweenness centrality algorithm was applied, measuring the number of shortest paths going through a node for a given network. This takes into consideration the global network structure, rather than the immediate neighbor of the query node (35).

To identify pathways enriched from input metabolites, MSEA was implemented with hypergeometric testing through over-representation analysis (ORA) using the KEGG database with 80 registered Human metabolic pathways (34). Enriched pathways p-values were subject to FDR correction and presented with relative impact values. Impact values were calculated autonomously through pathway topology and presented as a cumulative percentage representing the importance of all matched metabolites for the enriched query pathway (60).

## Results

### Baseline characteristics of the cohorts

All 24 women enrolled completed the three treatment arms. A subset of 20 women had MRI-assessed IPFD, and a range of 2.13 to 12.7% IPFD was calculated. Mean (SD) age, BMI, FPG and IPFD are presented for both ethnicity (European Caucasian and Asian Chinese) and IPFD (Low and High) cohorts (**Table 1**). In comparison to the Caucasian cohort (n=12), the Chinese cohort (n=12) had a significantly lower age and BMI, but similar FPG and IPFD. When comparing IPFD cohorts, the High IPFD cohort (n=10) had a significantly higher mean age, but similar BMI and FPG to the Low IPFD cohort.

**Table 1:** Participant characteristics.

	Ethnicity			Intra-pancreatic fat		
	Caucasian (n=12)	Chinese (n=12)	P value	Low (n = 10)	High (n = 10)	P value
**Age (years)**	54.7 ± 15.6	42.0 ± 10.8	0.038	35.9 ± 12.5	53.9 ± 8.1	0.002
**BMI (kg/m^2^)**	31.4 ± 4.2	26.9 ± 3.8	0.014	28.7 ± 3.8	28.2 ± 5.1	0.803
**FPG (mmol/L)**	6.1 ± 0.4	5.9 ± 0.3	0.257	5.8 ± 0.4	6.1 ± 0.4	0.147
**IPFD (%)**	5 ± 1.5*	4.8 ± 2.4	0.864	3.5 ± 0.8	6.2 ± 2.1	0.002

Values are mean ± standard deviation (SD). * n=8; MRI artifact resulted in missing values for 4 individuals. BMI, body mass index. FPG, fasting plasma glucose. IPFD, intra-pancreatic fat deposition; % pancreas fat. Statistical significance set at P < 0.05

### Postprandial metabolome responses

In total, 524 features (positive and negative ionization mode) were detected by HILIC-MS metabolomics. After batch correction, filtration and removal of noise, a matrix of 367 features was subject to linear mixed effect modeling to determine potential metabolites impacted by WPI intake over time, accounting for age and BMI. 216 features were significant (q-value ≤ 0.01) for Meal*Time interaction and were further processed as incremental area under the curve (iAUC) per meal and fold change (FC) calculated between meals 0 g WC/12.5 g LP and 0 g WC/50 g HP respectively (**Supplementary Figure 1** **).** FC values identified 92 up-regulated and 8 down-regulated features with the consumption of 12.5 g LP. After 50 g HP, 14 additional features were up-regulated, 11 features were down-regulated relative to 12.5 g LP, leaving a total of 125 features of interest.

### Ethnicity and intra-pancreatic fat deposition modelling

**Figure 1:**
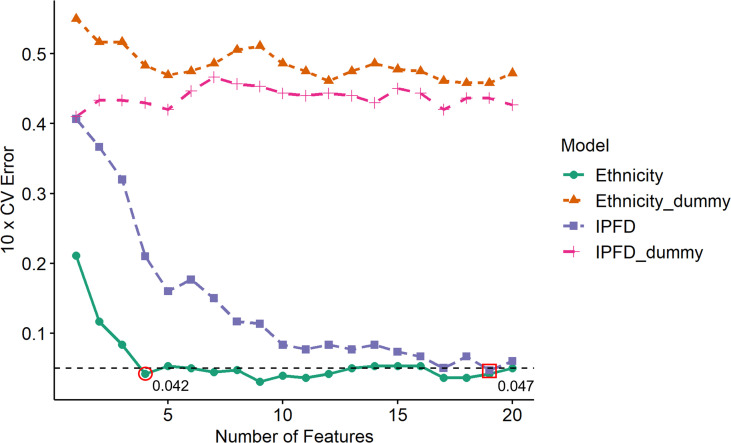
Generalized ten-fold cross validation error estimates for each testing set iteration per number of top SVM-RFE input features. Ten-fold CV error estimates depict the prediction accuracy of ethnicity and intra-pancreatic fat deposition (IPFD) models relative to number of top-ranking features input at each testing fold iteration. Ethnicity_dummy and IPFD_dummy models represent respective query model classes against shuffled y axis values (feature vectors), reflecting the classification legitimacy of each query model. Horizontal dotted line indicates threshold for optimal features input for an acceptable error estimate value (< 0.05) for model prediction. Red circle denotes the minimum number of input features to obtain an optimal error estimate to classify Ethnicity as a model. Red square denotes the minimum number of input features to obtain an optimal error estimate to classify IPFD as a model.

Support vector machine-recursive feature elimination (SVM-RFE) in combination with 10-fold cross-validations was implemented to classify Ethnicity (Caucasian and Chinese; n=24) and IPFD (Low and High; n=20) models with the mass spectrometer features identified in the postprandial whey protein response. Both Ethnicity and IPFD SVM-RFE models with respective Ethnicity_dummy and IPFD_dummy confusion models were plotted to compare their relative success in classification by the number of top-ranking features relative to generalized error estimates (**Figure 1**). Neither dummy model identified an optimal generalized error estimate with any given number of input features, while Ethnicity as a model was classified with an error estimate of 0.042 from the input of four top-ranking features, and IPFD was classified with an error estimate of 0.047 from the top 19 features. Therefore, the top 20 features were annotated discriminating both Caucasian and Chinese cohorts and Low and High IPFD cohorts.

The input of imidazolelactic acid, uric acid, *N*(ϵ)-methyl-lysine and L-cystine as model metabolites alone was sufficient in discriminating Caucasian and Chinese cohorts (**Table 2** **)**. A closer look at the top-ranking features for Ethnicity by plotting Time and Meal identified Caucasians as having a base (T=0) two-fold higher separation of imidazolelactic acid and *N*(ϵ)-methyl-lysine levels than the Chinese cohort, regardless of WPI (**Supplementary Figure 2A, 2C**). While both models had citric acid, creatine, glycine, imidazolelactic acid, *N*(ϵ)-methyl-lysine, octopamine, ornithine and uric acid as top-ranking metabolites (**Table 2**), only the Ethnicity model presented branched-chain amino acids valine, isoleucine and leucine within the classification model. The top IPFD metabolite, Uric acid, presented an average ranking score of 4.1 across all testing folds, higher than the ranking scores of top four ranking Ethnicity metabolites (2 - 3.8). The smaller the average ranking score for a feature, the greater its contribution towards a SVM classification. This indicates that the features significant for Meal*Time interaction had a greater strength for predicting ethnicity as a model than IPFD, and that uric acid is a stronger predictor of ethnicity than it is for IPFD.

**Table 2:** Top 20 SVM-RFE metabolites for predicting ethnicity and IPFD models.

Ethnicity model			IPFD model		
Annotation	AvgRank	Meal*Time	Annotation	AvgRank	Meal*Time
Imidazolelactic acid	2	6.56E-03	Uric acid	4.1	2.40E-03
Uric acid	2.6	2.40E-03	N(ϵ)-Methyl-Lysine	9.4	6.89E-04
N(ϵ)-Methyl-Lysine	2.7	6.89E-04	Octopamine	12.8	6.89E-04
L-Cystine	3.8	2.81E-02	Unk m/z 156.895: rt 762s	13.2	1.41E-02
Glycine	6.5	6.89E-04	L-Lysine	17.8	6.89E-04
L-Valine	14.8	6.89E-04	Ornithine	19.5	6.89E-04
L-Arginine	15.1	6.89E-04	L-Tyrosine	20.6	6.89E-04
Citric acid	19.1	6.89E-04	Imidazolelactic acid	21.7	6.56E-03
Octopamine	19.9	6.89E-04	Glyceric acid	22.1	4.53E-03
Ornithine	21.9	6.89E-04	L-Glutamine	23.7	1.30E-03
4-Aminobutanoate	23.3	1.07E-02	L-Histidine	25.3	6.89E-04
L-Methionine	23.5	6.89E-04	Phenylalanyl-valine	25.3	6.89E-04
L-Asparagine	25.6	6.89E-04	2-Aminobutyrate	27.5	6.89E-04
Creatine	25.6	6.89E-04	L-Threonine	29.4	6.89E-04
Urea	26.2	6.89E-04	Glycine	29.9	6.89E-04
Unk m/z 199.038: rt 591s	27.1	4.53E-03	Creatine	30	6.89E-04
L-Isoleucine	27.1	6.89E-04	3-Oxopentanoic acid	30.8	4.53E-03
Leucine	27.2	6.89E-04	Citric acid	31.8	6.89E-04
L-Glutamic Acid	29.4	6.89E-04	Unk m/z 778.516: rt 650s	37.8	1.30E-03
L-Phenylalanine	32.7	6.89E-04	L-Serine	44.2	6.89E-04

AvgRank values represent each features average ranking over all cross-validation folds of the kernel function SVM-RFE. Smaller values indicate better classification towards the query model. Meal*Time represents the calculated FDR corrected permutation p-value for the metabolite in the LME model. Unk, Unknown metabolites are denoted by m/z, mass charge and rt, retention time in seconds. Shaded metabolites were recognised within the metabolic network and pathway enrichment databases.

**Figure 2:**
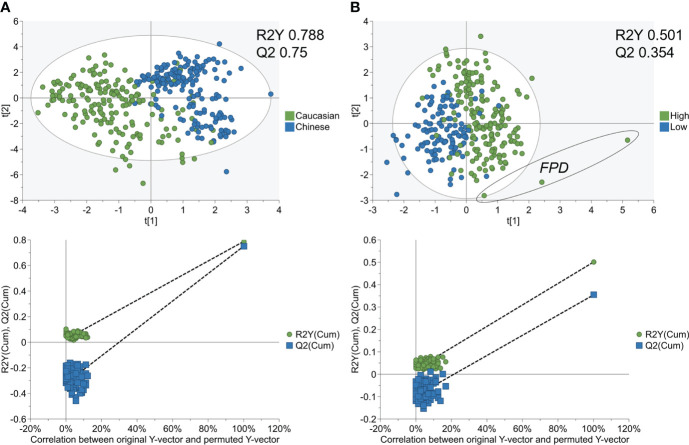
PLS−DA analysis of SVM-RFE metabolome models for Ethnicity cohorts and IPFD classes. PLS−DA score plot (top) and 100 permutation tests (bottom) showing **A**) good separation and robust model for SVM-RFE ranked metabolites between Caucasian and Asian Chinese, and **B**) moderate separation and predictive modelling for SVM-RFE ranked metabolites between Low and High IPFD. FPD, fatty pancreas disease > 10.4% IPFD).

The strength of both SVM-RFE models was further validated through PCA and PLS-DA. While Ethnicity PCA modelling (R2X 0.526) had better separation than IPFD (R2X 0.428), both were low in separation as an un-supervised model from their respective cumulative R2X values (Q2 0.297, Q2 0.284) (**Supplementary Figures 4A, 4B**). PLS-DA resulted in a strong separation of Ethnicity with permutation testing (R2Y 0.788, Q2 0.75) (**Figure 2A**), while the IPFD model PLS-DA presented a moderate separation with ranking features (R2Y 0.501, Q2 0.354). (**Figure 2B**)

### Metabolic network analysis of ethnicity model metabolites

**Figure 3:**
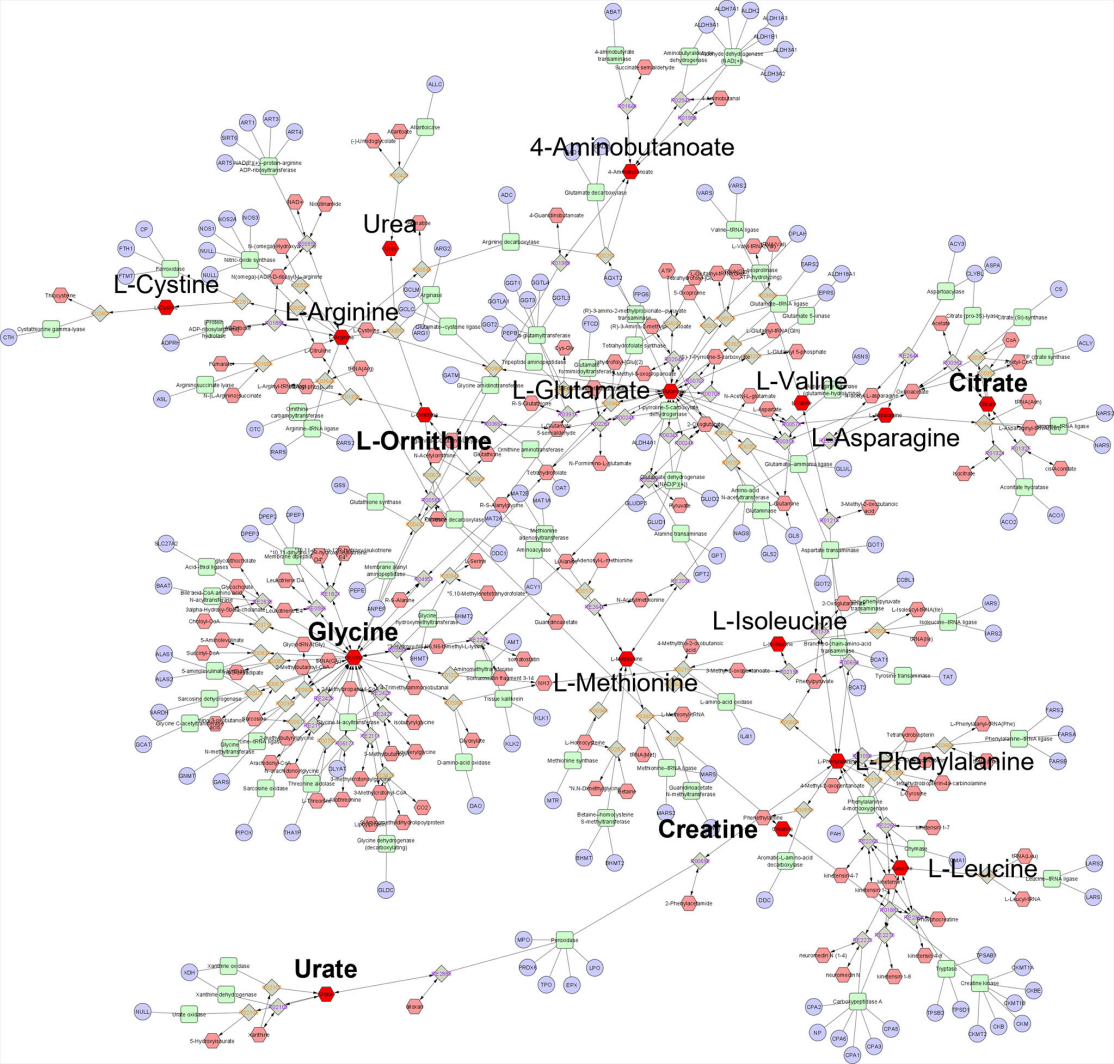
Global metabolic network pathways identified with top ranking Ethnicity model metabolites. Nodes correspond to an identified KEGG compound/gene/reaction, and edges indicate a significant correlation between nodes. Annotated red hexagons represent input metabolites, pink hexagons represent network metabolites, blue circles represent encoding genes, green quadrangles represent associated enzymes and grey diamonds represent metabolic reactions. Metabolites in bold are hub metabolites by degree and betweenness centrality measures.

From the top-ranking metabolites discriminating participants Ethnicity by postprandial WPI response; 19 metabolites presented KEGG IDs, in which 16 (**Table 2**) were available for metabolic network analysis and pathway enrichment when using the Metscape and KEGG databases against 80 human metabolism pathways. Ethnicity model metabolites constructed a single component metabolic network with 492 nodes and 563 edges (**Figure 3**). Network centrality identified glycine, L-glutamate, and L-phenylalanine as hub nodes among the array of metabolites, enzymes and genes based on their measure of degree (>10) and betweenness centrality (>0.1) (**Table 3**). Over representation analysis (ORA) identified three pathways as significant with 16 ethnicity associated metabolites (**Table 4**), This included alanine, aspartate and glutamate metabolism with four metabolites, arginine biosynthesis with four metabolites, and arginine and proline metabolism with five metabolites.

**Table 3:** Topological parameters of key metabolites classifying ethnicity as an SVM-RFE model.

Metabolite	Degree	Betweenness centrality	Pathways
Glycine	27	0.4155	Bile acid biosynthesis. Glycine, serine, alanine, and threonine metabolism. Leukotriene metabolism. Lysine metabolism. Porphyrin metabolism. Urea cycle and metabolism of arginine, proline, glutamate, aspartate, and asparagine. Vitamin B9 (folate) metabolism.
L-Glutamate	23	0.4274	Histidine metabolism. Urea cycle and metabolism of arginine, proline, glutamate, aspartate, and asparagine. Vitamin B9 (folate) metabolism.
L-Phenylalanine	11	0.2803	Tyrosine metabolism. Biopterin metabolism.

The degree of a node is the number of edges associated with it, and the betweenness centrality of a node is the number of shortest communication paths between different pairs of nodes.

**Table 4:** Metabolic pathway enrichment for ethnicity WPI metabolome response model.

Pathway name	Total compound	Hits	p.Adjusted	Impact
Arginine and proline metabolism	38	5	3.77E-03	0.2905
Alanine, aspartate, and glutamate metabolism	28	4	0.018	0.2837
Arginine biosynthesis	14	4	9.87E-04	0.2538

WPI, whey protein isolate. ‘Total compound’ represents the number of compounds involved in the pathway. ‘Hits’ represents the matched number from the input query metabolites. ‘p.Adjusted’ represents the original p-value calculated from the enrichment analysis adjusted with Holm-Bonferroni corrections. ‘Impact’ represents the cumulative percentage of importance from pathway topology analysis comparing different pathways.

### Metabolic network analysis of intra-pancreatic fat deposition model metabolites

**Figure 4:**
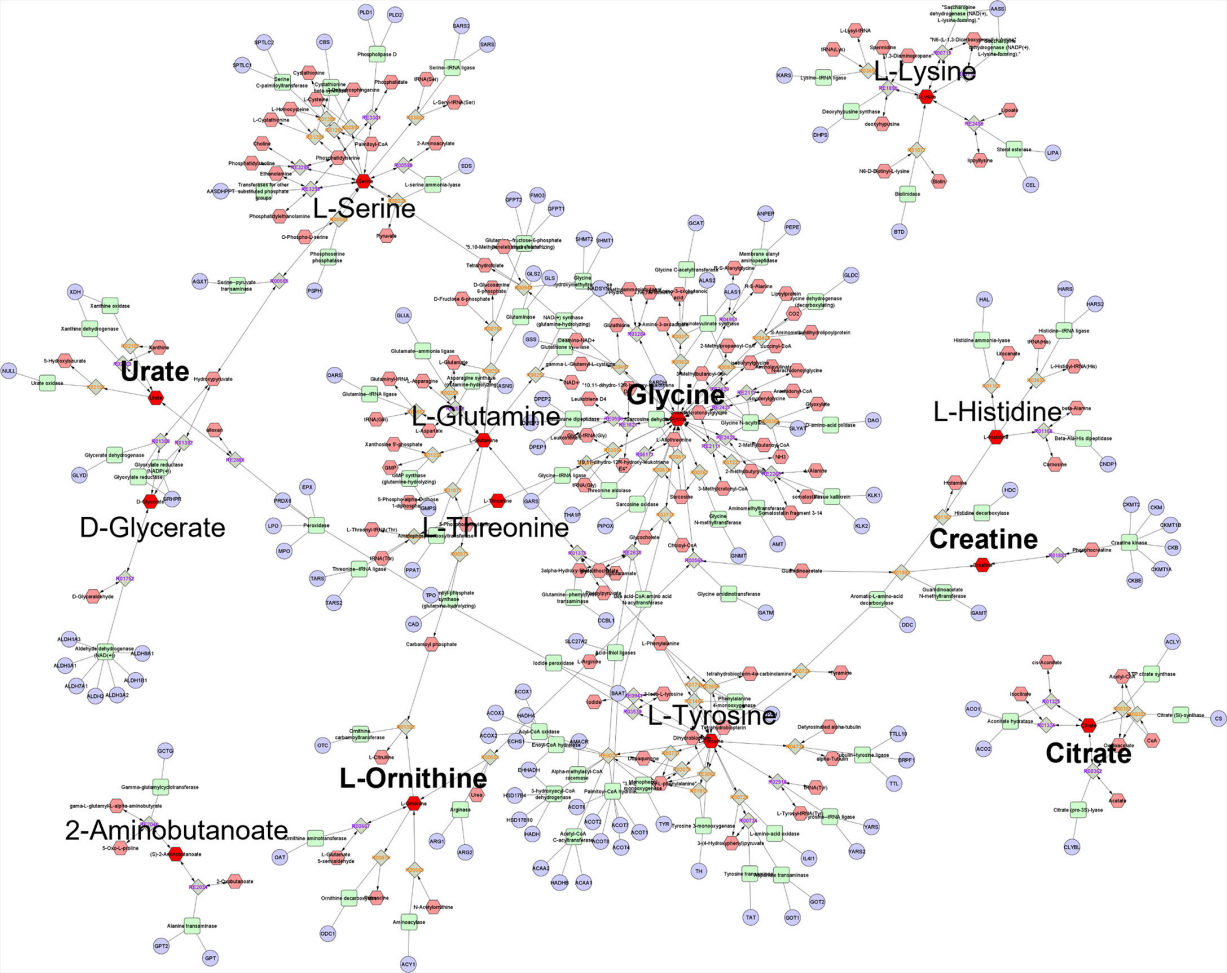
Global metabolic network pathways identified with top ranking IPFD model metabolites. Nodes correspond to an identified KEGG compound/gene/reaction, and edges indicate a significant correlation between nodes. Annotated red hexagons represent input metabolites, pink hexagons represent network metabolites, blue circles represent encoding genes, green quadrangles represent associated enzymes and grey diamonds represent metabolic reactions. Metabolites in bold are hub metabolites by degree and betweenness centrality measures.

Of the 20 top-ranking postprandial WPI response metabolites separating participants with low IPFD from high IPFD, 16 had KEGG IDs, of which 13 (**Table 2**) were processed for pathway enrichment and metabolic network construction. IPFD model metabolites constructed a four-component metabolic network with 454 nodes and 504 edges (**Figure 4**). Network centrality identified glycine, L-tyrosine, L-serine, and L-glutamine as hub nodes based on their measure of degree (>10) and betweenness centrality (>0.1) (**Table 5**). ORA identified three pathways as significant with 13 IPFD metabolites (**Table 6**), This included glycine, serine, and threonine metabolism with five metabolites, glyoxylate and dicarboxylate metabolism with five metabolites, and aminoacyl-TRNA biosynthesis with seven metabolites.

**Table 5:** Topological parameters of key metabolites classifying IPFD as an SVM-RFE model.

Metabolite	Degree	Betweenness centrality	Pathways
Glycine	27	0.705	Bile acid biosynthesis. Glycine, serine, alanine, and threonine metabolism. Leukotriene metabolism. Lysine metabolism. Porphyrin metabolism. Urea cycle and metabolism of arginine, proline, glutamate, aspartate, and asparagine. Vitamin B9 (folate) metabolism.
L-Tyrosine	15	0.4699	Tyrosine metabolism. Biopterin metabolism.
L-Serine	13	0.3081	Glycerophospholipid metabolism. Glycine, serine, alanine and threonine metabolism, Glycosphingolipid metabolism. Methionine and cysteine metabolism. Vitamin B9 (folate) metabolism.
L-Glutamine	10	0.2181	Amino sugars metabolism. Purine metabolism. Pyrimidine metabolism. Urea cycle and metabolism of arginine, proline, glutamate, aspartate, and asparagine. Vitamin B3 (nicotinate and nicotinamide) metabolism.

The degree of a node is the number of edges associated with it, and the betweenness centrality of a node is the number of shortest communication paths between different pairs of nodes.

**Table 6:** Metabolic pathway enrichment for IPFD WPI metabolome response model.

Pathway name	Total compound	Hits	p.Adjusted	Impact
Glycine, serine and threonine metabolism	33	5	9.71E-04	0.487
Glyoxylate and dicarboxylate metabolism	32	5	8.39E-04	0.2593
Aminoacyl-tRNA biosynthesis	48	7	1.36E-05	0.1667

Total compound is the number of compounds involved in the pathway. Hits is the matched number from the input query metabolites. p.Adjusted is the original p-value calculated from the enrichment analysis adjusted with Holm-Bonferroni corrections. Impact value is the cumulative percentage of importance from pathway topology analysis comparing different pathways.

## Discussion

To the best of our knowledge, this is the first study to model the postprandial WPI response to determine differences in metabolomic profiles between ethnic groups and/or groups with various levels of IPFD. It is also the first reported comparison of the postprandial metabolome responses associated with IPFD and ethnicity as an estimate of prediabetic risk factors. While most postprandial response studies measure differences associated with beverage composition or the capacity of a beverage to elicit a response (27, 48, 61, 62), this study utilized prior knowledge of WPI response as a basis for discerning differences between cohorts likely not apparent when comparing only basal metabolite levels.

Characterizing a metabolic response to a dietary intervention can determine an individual’s risk of developing a disease outcome (63). We hypothesized that differences in postprandial WPI response could provide insight into how IPFD and/or ethnicity may contribute to adverse metabolic health outcomes. Therefore, we measured the postprandial response to WPI in a cohort of overweight women with prediabetes using untargeted metabolomics. Our aim was to discern differences in ethnicity- and IPFD-associated biomarkers following WPI intake. Notably, in the TOFI_Asia study, using PLS-DA analysis we had previously identified a clear and robust separation in fasting lipid and polar metabolite profiles that characterized the two ethnicity cohorts (19). In our current data set we confirmed this separation through fasting polar metabolites; 3-methoxytyrosine, dihydrothymine, asymmetric dimethylarginine, valeric acid, 1-methyl-L-histidine and succinic acid. Students t-test analysis of these plasma metabolites identified a significant difference at baseline (T=0) between ethnicity cohorts, and no temporal response to increasing doses of WPI. Also of note, we had previously identified dihydrothymine, valeric acid and 1-methyl-L-histidine as significantly different between ethnicity cohorts in the larger data set of Asian Chinese and European Caucasian men and women from the TOFI_Asia study (19). The difference in basal level metabolites between Caucasian and Chinese cohorts indicates a clear disparity in metabolic pathways. Although these metabolites may contribute to resistance or susceptibility to developing T2D, they were omitted from the postprandial WPI models due to their lack of temporal profile.

We developed a feature selection pipeline that first identified a set of relevant features by linear mixed effect (LME) modeling for Meal*Time interactions with the inclusion of participants age and BMI, then removing LME false positives by incremental area under the curve-fold change (iAUC-FC) analysis, then comparing each participant WPI concentration dependent response to their respective postprandial water control response. Resulting features were used to model the differences in WPI response associated with ethnicity (Caucasian and Chinese) or IPFD (low IPFD and high IPFD) classes through a SVM-RFE algorithm. SVM-RFE found that the postprandial WPI metabolome response was most impacted by differences associated with ethnicity rather than IPFD, as average ranking values for metabolites classifying ethnicity as a model were greater (lower in value) than those for IPFD modelling. Additionally, the ethnicity model required far less metabolites to discriminate Caucasian and Chinese participants, than participants with low or high IPFD. The discriminant power of these models was confirmed by PLS-DA, where a strong separation between Caucasian and Chinese participants was achieved, in agreement with our previous data from the TOFI_Asia study (19), while only a moderate separation of IPFD classes from SVM-RFE ranking metabolites was produced. We set a threshold of ≥ 4.66% as cut point for high IPFD, based on the cohort median, which is 0.16% higher than the weighted mean obtained in a 2017 meta-analysis (38). A value of 10.4% for IPFD has previously been proposed as the cut point for fatty pancreas disease (FPD) by Wong et al. (22), based on a large cohort of Hong Kong Chinese. Whilst differences in MRI techniques and post scan analysis methods prevents robust between-study comparison, we note contribution of one participant in our current cohort with an IPFD value of 12.17% which may represent FPD, and which is presented as a strong outlier in the IPFD PLS-DA.

Network topology and metabolite enrichment set analysis (MESA) in our current cohort provided a weighted evaluation of discriminatory metabolites by their annotated function in human metabolism; characterizing key network hubs and enriched metabolic pathways that separate both Caucasians and Chinese participants, and low IPFD from high IPFD participants by WPI metabolome response. While both models enriched different metabolic pathways, central to each ethnicity- and IPFD-WPI response network was the amino acid glycine. Considered a semi-essential amino acid, glycine has a key role in many metabolic pathways, including protein biochemistry, nitrogen metabolism, bile acid conjugation, and central nervous system signaling as a neurotransmitter (64). Genome wide-association studies (GWAS) using both single-nucleotide polymorphisms (SNPs) and exome sequencing data have linked plasma glycine levels to genetic variants in the carbamoyl phosphate synthase 1 (CPS1) gene, which encodes the rate-limiting step of the urea cycle (48, 65). This aligns with our network topology analysis, which identified the urea cycle as a key metabolic subnetwork from discriminatory metabolites for ethnicity. This included glycine and glutamic acid as key network hubs, and urea, arginine, asparagine, glutamic acid, ornithine, and 4-aminobutyric acid as contributing urea network nodes. The most impacted pathway discriminating Caucasians from Chinese was arginine and proline metabolism which is fundamental to urea production from arginine *via* arginase-1 activity (66). The representation of urea cycle metabolites among the discriminatory model for ethnicity indicates differences in postprandial ammonia and nitrogen metabolism pathways between Caucasians and Chinese presented with a WPI challenge.

A closer look at the glycine profiles within ethnicity and IPFD cohorts found a prominent depletion of levels relative to increasing WPI concentration. Though the WPI beverages contained trace amounts of glycine (0.2 – 0.9 g) (39), it was apparent that high IPFD participants fed with 12.5 g of WPI were more sensitive to glycine depletion than participants with low IPFD. Glycine depletion was also more pronounced in the Chinese cohort’s response to 50 g or 12.5 g WPI than Caucasians. These results contrast other postprandial studies, wherein men or women with a BMI within the healthy range (≤ 25 kg/m^2^) had increased levels of glycine in response to a protein supplement (48, 65). Interestingly, the BCAAs; valine, leucine, and isoleucine, were identified as top-ranking metabolites for the discrimination of ethnicity as a WPI response in women with prediabetes. Increased BCAAs have been positively associated with insulin resistance, diabetic nephropathy, and dyslipidemia in epidemiological studies (67, 68). Furthermore, glycine levels have been negatively associated with metabolic syndrome, obesity, and diabetic complications (69–71). By use of the Zucker-fatty rat (ZFR) and Zucker-lean rat (ZLR) models, White et al., demonstrated that the raised levels of BCAAs associated with obesity generates excess levels of ammonia from increased BCAA transamination, leading to the recruitment of glycine as a carbon donor for the pyruvate-alanine cycle (72). Our results indicate that the TOFI phenotype contributes towards the depletion of glycine more so than BMI, as the mean BMI of both IPFD cohorts was not significantly different, but their glycine response differed. The Chinese cohort, with a mean BMI of 26.9 ± 1.1 had greater sensitivity to glycine depletion than the Caucasian cohort, whose mean BMI of 31.4 ± 1.32 was significantly greater.

Although the IPFD model presented glycine within the top-ranking metabolites, it lacked the ranking of BCAAs as seen within the ethnicity model. Instead, the IPFD model ranked the aromatic amino acid tyrosine, whose presence has been an established biomarker for the exacerbation of insulin dysregulation in patients with non-alcoholic fatty liver disease (NAFLD) and diabetic nephropathy (73–76). Interestingly, the most impacted pathway discriminating low IPFD from high IPFD participants was glycine, serine, and threonine metabolism, as serine and threonine are key metabolites involved in the *de novo* synthesis of glycine (64), and along with glutamine, which was also ranked with high IPFD, are all associated with purine metabolism and the formation of excess uric acid (64, 77, 78). Uric acid was the top-ranking metabolite discriminating low IPFD participants from high IPFD through their WPI metabolome response. Commonly associated with Gout formation in joints, uric acid has long been considered as an inert end-product from purine degradation (79). But recent studies have shed light on uric acid as a regulator of key metabolic signaling pathways; stimulating fat storage and insulin resistance through adenosine monophosphate (AMP) deaminase, or promoting fat degradation and the decrease of gluconeogenesis through AMP activated protein kinase (80–82). While these attributes were once advantageous during times of food scarcity, it has been hypothesized that they have become detrimental to modern humans who lack a functional urate oxidase enzyme, resulting in higher levels of serum uric acid during an era of obesity (83). With a decrease in both glycine and serine in response to WPI, along with increased levels of plasma uric acid in high IPFD participants, an inappropriate signal of fat storage and insulin resistance could be perpetuated towards further metabolic complications.

Two metabolites impacted by WPI that significantly contributed towards both SVM-RFE models, in particular the separation of Caucasians from Chinese participants, were imidazolelactic acid and *N*(ϵ)-methyl-lysine. Imidazolelactic acid is formed from the reduction of imidazole-pyruvate, which represents a key branch point in the source production of aspartate from the histidine transaminase pathway in *Escherichia coli* (84). *N*(ϵ)-methyl-lysine is a poorly characterized metabolite, first detected in small concentrations by chromatographing plasma from fasting humans (85, 86). Production of *N*(ϵ)-methyl-lysine has been reported in *Proteus vulgaris* bacteria (87). Both metabolites were higher at basal level within the Caucasian cohort, with imidazolelactic acid decreasing in response to WPI concentrations and *N*(ϵ)-methyl-lysine increasing in response to WPI. Due to their unique profile of complete separation at basal level, but with a postprandial WPI response, and absence from the human metabolic pathway database, we speculate that they are associated with ethnic differences relating to gut microbiota profiles, as both *Escherichia coli* and *Proteus vulgaris* bacteria have been associated with the human gastrointestinal microbiome previously (88).

In conclusion, our study used untargeted metabolomics and postprandial WPI responses to identify a set of metabolites both common and disparate between IPFD and ethnicity models, using SVM-RFE modelling in overweight women with prediabetes. The discriminant power of these models demonstrated a strong separation of metabolites between European Caucasian and Asian Chinese participants, in agreement with our prior data from the TOFI_Asia study. Network analysis and pathway enrichments revealed several metabolites of the urea cycle, and arginine and proline metabolism that could differentiate between Caucasian and Chinese participants. Previously we identified a strong association of creatine for the Chinese cohort in our larger TOFI_Asia study, which was further validated in this study as a contributing metabolite for the discrimination of both ethnicity and IPFD WPI metabolome response models. Metabolites of the glycine, serine, and threonine metabolism were used in the discrimination of low and high IPFD classes, therefore implicating purine synthesis and uric acid production with increased IPFD levels. Betweenness centrality identified glycine as a key network hub for both ethnicity and IPFD metabolome networks, representing a difference in contribution towards urea cycle and uric acid metabolism, respectively. Glycine depletion was most prominent in the Chinese cohort relative to the Caucasian cohort, the latter notably with significantly higher BMI. Furthermore, the high IPFD cohort had a more prominent glycine depletion profile than the low IPFD cohort, despite comparable BMI. These results further characterize the obesity associated postprandial glycine profile established in the literature, but in addition brings to light the relative contribution that VAT and ectopic fat deposition have over BMI as an exacerbator of glycine depletion in a cohort with impaired fasting glucose. This study identified several unknown features as potential metabolites, annotated by their respective retention time and mass charge. These and other metabolites within this study, such as imidazolelactic acid and *N*(ϵ)-methyl-lysine, will need to be further characterized before they can be considered for systems biology modelling in future cohorts.

## Data availability statement

The datasets presented in this study can be found in the EMBL-EBI MetaboLights database with the identifier MTBLS5568. You can access the study here: https://www.ebi.ac.uk/metabolights/MTBLS5568.

## Ethics statement

This study was reviewed and approved by Auckland Health and Disabilities Ethics Committee (HDEC, Reference: 17/NTA/172), New Zealand. Study was registered with the Australia New Zealand Clinical Trial Registry (ANZCTR, Reference: ACTRN12618000145202. The patients/participants provided their written informed consent to participate in this study.

## Author contributions

All listed authors meet the requirements for authorship. IS led the clinical part of this study. IS, WY, JL and LL contributed to participants recruitment, sample collection and the clinical data. IS, and WY contributed to IPFD measurements. KF led and supervised the metabolomics part of this study. ZW conducted sample extraction and metabolomics profile acquisition. BJ and KF advised on statistical analysis. AJ-M conducted data analyses, results interpretation and drafted the manuscript. IS, JL, BJ, KF and SP critically revised and commented on the first and subsequent drafts. SP was the principal investigator for the Metabolic Health platform within the National Science Challenge High Value Nutrition (HVN) program, and fundraiser, who conceptualized and designed this study. All authors contributed to the article and approved the submitted version.

## Funding

This study was funded by the New Zealand National Science Challenge High Value Nutrition Program, Ministry for Business, Innovation and Employment (MBIE), Grant # 3710040.

## Acknowledgments

Fonterra Co-operative Group Ltd, New Zealand provided the whey protein isolate for this intervention.

## Conflict of interest

The authors declare that the research was conducted in the absence of any commercial or financial relationships that could be construed as a potential conflict of interest.

## Publisher’s note

All claims expressed in this article are solely those of the authors and do not necessarily represent those of their affiliated organizations, or those of the publisher, the editors and the reviewers. Any product that may be evaluated in this article, or claim that may be made by its manufacturer, is not guaranteed or endorsed by the publisher.
