# Supplementary material for: The impact of ethnicity and intra-pancreatic fat on the postprandial metabolome response to whey protein in overweight Asian Chinese and European Caucasian women with prediabetes
Source: Front Clin Diabetes Healthc. 2022 Oct 14;3:980856. doi: 10.3389/fcdhc.2022.980856 (PMC10012149; doi:10.3389/fcdhc.2022.980856)
Supplement: Supplementary file 1 [file DataSheet_1.docx]

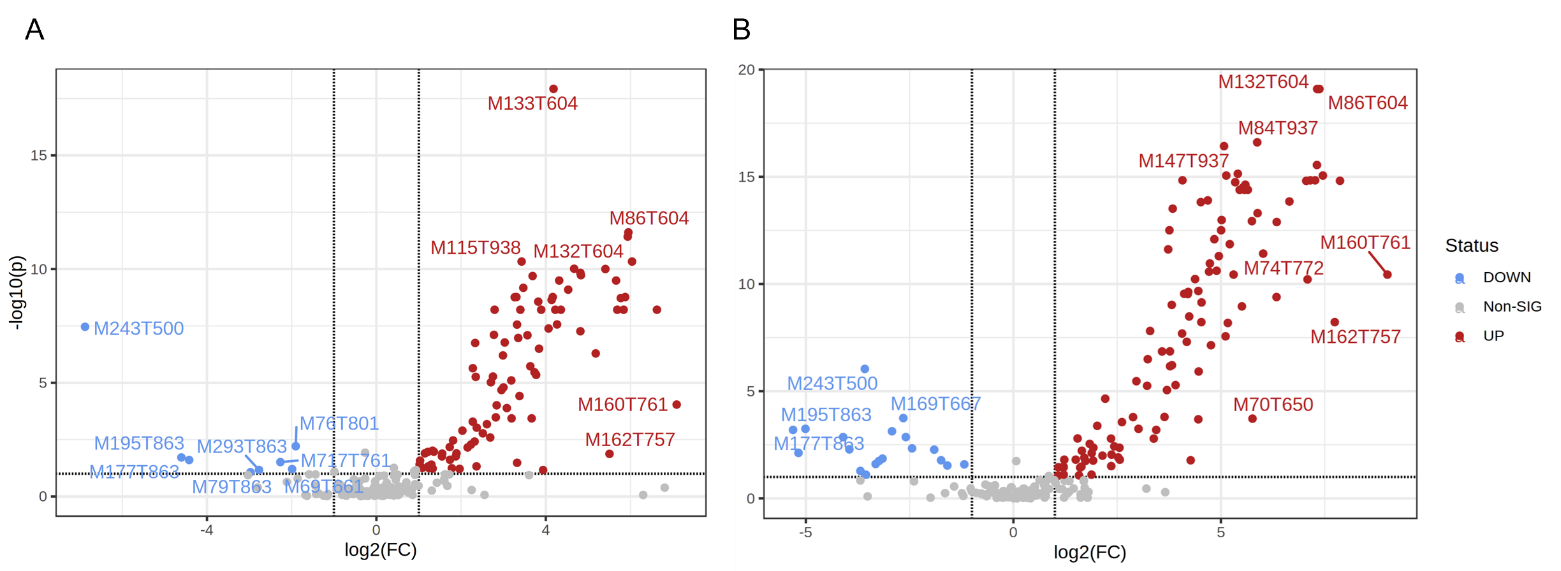


**Supplementary Figure 1:** **A**) Fold-change calculations comparing AUC values of 12.5g and **B**) 50g whey protein intake relative to 0g water intake. FDR corrected features selected by volcano plot with fold change threshold (x) 2 and t-tests threshold (y) 0.1. The red circles represent features above the threshold. Note both fold changes and p-values are log transformed. The further its position away from the (0,0), the more significant the feature is. Features are named by their mass-charge (M) and retention time (T)


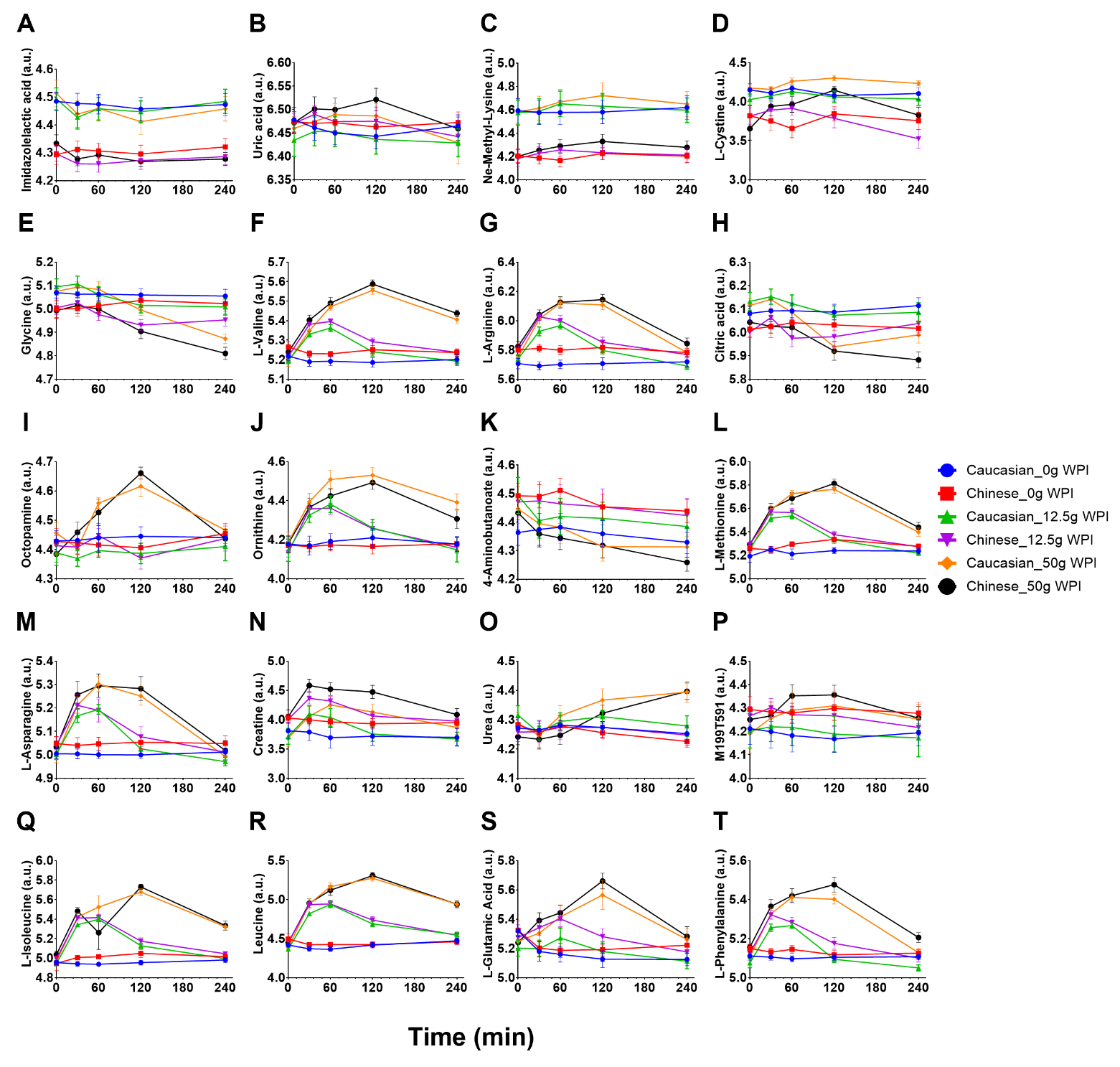


**Supplementary Figure 2:** Postprandial concentration of top SVM-RFE metabolites classifying Ethnicity. Mean (± SEM**) A**) Imidazolelactic acid, **B**) Uric acid, **C**) N(ε)-Methyl-Lysine, **D**) L-Cystine, **E**) Glycine, **F**) L-Valine, **G**) L-Arginine, **H**) Citric acid, **I**) Octopamine, **J**) Ornithine, **K**) 4-Aminobutanoate, **L**) L-Methionine, **M**) L-Asparagine, **N**) Creatine, **O**) Urea, **P**) M199T591, **Q**) L-Isoleucine, **R**) Leucine, **S**) L-Glutamic Acid, and **T**) L-Phenylalanine for each Ethnicity (Caucasian and Chinese) after ingesting water control (0g WPI), low protein (12.5g WPI) and high protein (50g WPI) at t = 0 min.


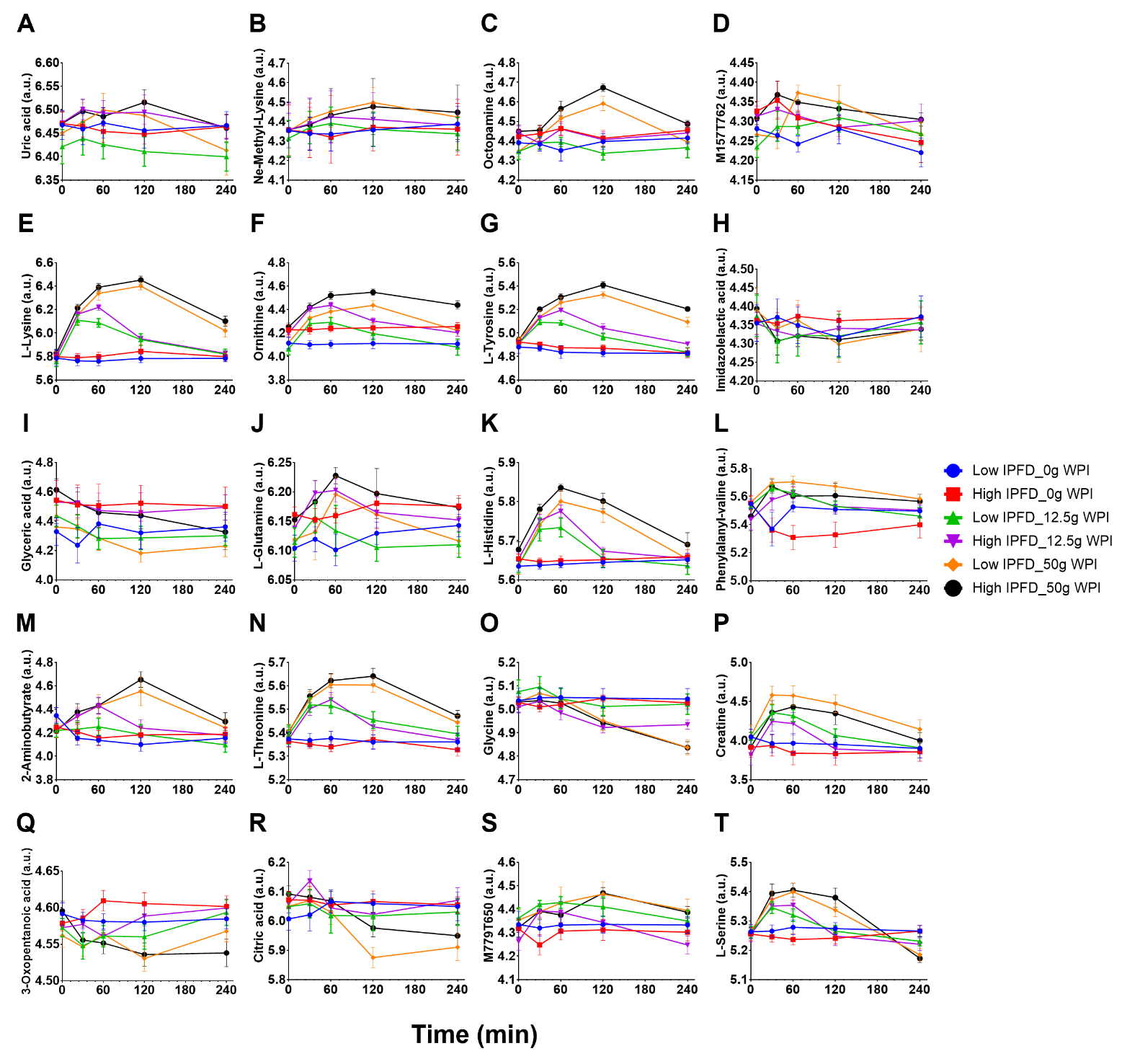


**Supplementary Figure 3:** Postprandial concentration of top SVM-RFE metabolites classifying Ethnicity. Mean (± SEM**) A**) Uric acid, **B**) N(ε)-Methyl-Lysine, **C**) Octopamine, **D**) M157T762, **E**) L-Lysine, **F**) Ornithine, **G**) L-Tyrosine, **H**) Imidazolelactic acid, **I**) Glyceric acid, **J**) L-Glutamine, **K**) L-Histidine, **L**) Phenylalanyl-valine, **M**) 2-Aminobutyrate, **N**) L-Threonine, **O**) Glycine, **P**) Creatine, **Q**) 3-Oxopentanoic acid, **R**) Citric acid, **S**) M779T650, and **T**) L-Serine for each IPFD class (Low and High) after ingesting water control (0g WPI), low protein (12.5g WPI) and high protein (50g WPI) at t = 0 min.

**
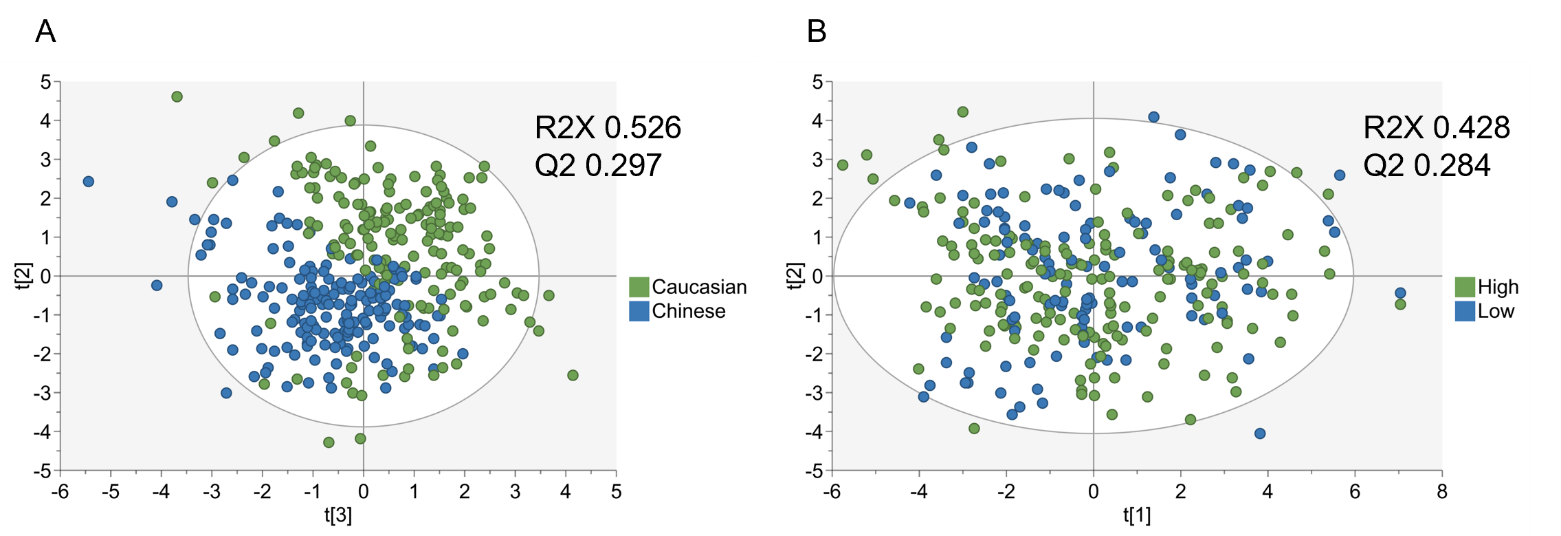
**

**Supplementary Figure 4:** **A**) PCA plot of the top 20 SVM-RFE ranked features for Ethnicity and **B**) Pancreatic Fat models. PCA models show the degree of unsupervised separation of respective model variables (Ethnicity:Caucasian and PanFat:High as green, Ethnicity:Chinese and PanFat:Low as blue) by designated components (t[ ]).
